# Reduction of noise pollution in CNC wood milling through multi-parameter optimization using response surface methodology

**DOI:** 10.1371/journal.pone.0332222

**Published:** 2025-12-15

**Authors:** Shiva Souri, Farshad Rabiei

**Affiliations:** 1 Department of Occupational Health and Safety Engineering, Faculty of Health, University of Medical Science, Ilam, Iran; 2 Department of Mechanical Engineering, Faculty of Engineering, Ilam University, Ilam, Iran; University of Vigo, SPAIN

## Abstract

**Background:**

CNC (Computer Numerical Control) wood milling machines offer significant productivity advantages but are associated with excessive noise pollution, posing health risks to workers. This study investigates the influence of machining parameters on Noise Pollution Level (NPL) in CNC wood milling and aims to optimize these parameters to minimize noise emissions.

**Methods:**

A Response Surface Methodology (RSM) based on Box-Behnken Design (BBD) was employed to model the effects of cutting speed, feed rate, depth of cut, and step over on NPL. A total of 27 experimental runs were conducted. Statistical analysis, including ANOVA and regression modeling, was performed to determine the significance of each parameter. The model was further optimized using a Genetic Algorithm (GA).

**Results:**

The NPL observed across experiments ranged from 97.4 dB to 103.8 dB, with all values exceeding the NIOSH recommended limit of 85 dB. ANOVA results revealed that cutting speed, cutting speed squared, feed rate, and depth of cut had a statistically significant effect on NPL (p  <  0.05). The regression model showed a high degree of fit (R²  =  0.945). Optimal parameters—cutting speed of 12,730 rpm, feed rate of 58 mm/s, depth of cut of 3.2 mm, and step over of 6.4 mm—were identified using GA, resulting in a predicted NPL of 96.2 dB, which closely matched the experimentally validated value of 95.8 dB.

**Conclusion:**

The study confirms that NPL in CNC wood milling can be significantly reduced by optimizing machining parameters. The integration of RSM and GA provides a reliable framework for minimizing occupational noise exposure, thereby enhancing worker safety in woodworking environments.

## 1. Introduction

CNC wood milling machines generate noise levels that not only exceed occupational safety thresholds (e.g., NIOSH’s 85 dB limit) but also contribute to long-term health issues like hearing loss [1]. Despite advances in CNC (Computer Numerical Control) technology, limited research has been conducted on mitigating noise pollution through optimization of machining parameters.

**Fig 1:**
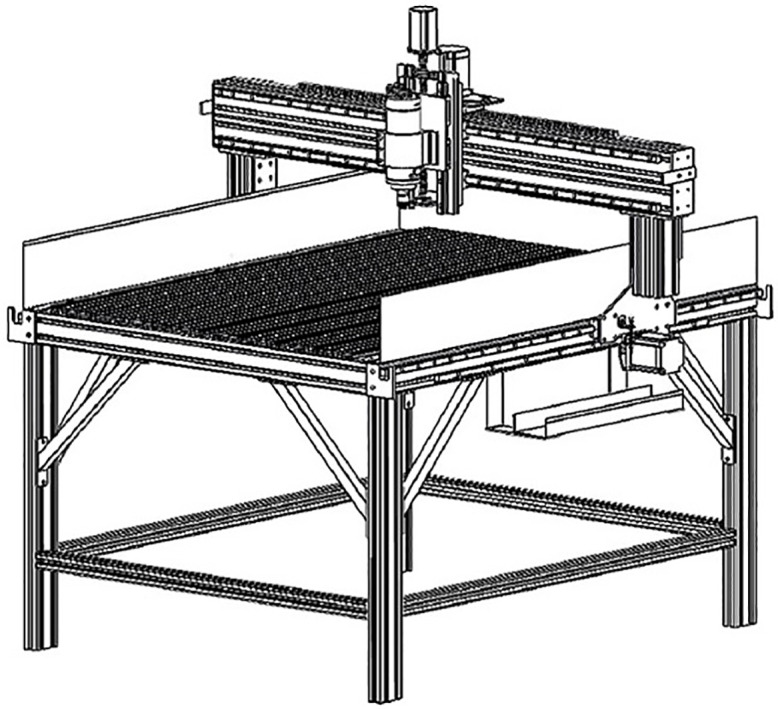
Three axis CNC machine.

Wood processing is one of the oldest manufacturing procedures, since wood is a fundamental material used in structures, apparatuses, furniture and musical instruments. In order to increase productivity and produce complex shapes, CNC machines are used (Fig 1). CNC machines are used in various manufacturing applications and are widely used in the woodworking industry [2]. In the furniture industry, CNC machines perform drilling, milling, sanding and cutting operations [3]. These machines provide high productivity increasing the efficiency and flexibility in production and integration to automation systems [ 4,5].

While prior studies have explored noise reduction in woodworking machinery, they have primarily focused on tool design and soundproofing. Few studies have investigated how machining parameters such as depth of cut, feed rate, cutting speed, and step over influence noise levels in CNC wood milling. This gap limits the development of eco-friendly and safe operating conditions for CNC machines. High noise levels may have negative impacts on the health and comfort of workers, and of the people living in the surroundings of a wood workshop [ 6,7]. Occupational hearing loss is one of the most widespread work-related illnesses in the world, which is caused by exposure of workers to high noise [8–11]. Also, industrial noise pollution leads to increase in blood pressure; increased stress; fatigue; vertigo; headaches; sleep disturbance; annoyance; speech problems; dysgraphia, which means reading/learning impairment; aggression; anxiety and withdrawal [12–14]. Based on information from WHO (World Health Organization), 6.1% of the world’s population (466 million people) were suffering from hearing loss in 2018. Also, this number will increase to 630 million people by 2030 and to more than 900 million by 2050 [15]. Exposure to hazardous noise is one of the physical agents of working with CNC milling that can first cause temporary hearing loss and then permanent hearing loss. The permanent hearing loss mainly starts from the frequency of 4000 Hz that cannot be corrected through surgery or with medicine [ 16,17]. NIOSH an 8-h time-weighted average exposure limit of 85 dB and a 3-dB exchange rate were recommended. Moreover, all workers exposed to noise levels above the recommended exposure limit (REL) are suggested to be entered in a hearing loss prevention program (HLPP) [ 18,19].

Limited research has been done in the field of noise pollution level (NPL) at wood working especially in case of in CNC milling. A study has been carried out to determine noise exposure, and to identify the extent of hearing damage among the wooden furniture industry workers from 30 selected factories, in the South East Asian region. Results revealed that 43% of the factories workers were exposed to the noise level higher than the recommended permissible limit, with 25.8% of them had a slight handicap with permanent threshold shift between 30 and 40 dB, while 8.9% of the workers showed a significant handicap with permanent threshold shift >40 dB [20].

Several studies have been done to reduce the noise of wood cutting machines, including: Spinelli et. al investigated the difference between the two main technology options (i.e., chippers and grinders) in order to offer additional decision elements to wood yard planners (in case of noise level). The chipper on test generated more noise than the grinder, due to its better ability to process wood and to transmit more energy into it. Since the chipper was equipped with less working tools and turned slower than the grinder, it generated its noise peaks at lower frequency bands [6]. Owoyemi et. al presented noise control techniques in wood working include; sound insulation, sound absorption, vibration damping and Vibration isolation [12].

Wellenreiter et. al investigated effect of tool specification on noise level. A conventional straight knife cutterhead and three helical knife cutterheads tested for planing black spruce wood (*Picea mariana*). Effects of helix angle and feed per knife (FK) on maximum cutting forces and sound level evaluated. Helical cutterheads considerably generated lower sound pressure level, with a maximum difference of up to 11.5 dB(A) [21].

On the other hand, many factors, including machining parameters, are effective on the amount of noise level. The control of machining parameters in all machining workshops can be adjusted by the operator and covers a wide range of woodworking industries. Therefore, in this research, the effect of machining parameters including depth of cut, feed rate, cutting speed and step over on the noise level of beech wood (solid wood) is evaluated. Based on the RSM, experimental tests are designed, then using the developed regression equation and analysis of variance, the effect of machining parameters on the amount of noise in beech wood is investigated and finally by optimization of regression equation, optimum condition is derived.

## 2. Materials and methods

To investigate the amount of NPL, four machining parameters (depth of cut (apa_pap), feed rate (vWv_WvW), cutting speed (vcv_cvc), and step over (sos_oso)) were examined. This study was conducted in a real industrial environment, i.e., a small workshop with only one CNC machine operating. Therefore, the environmental conditions of this research are representative of real industrial settings, but not of large factories with multiple machines operating simultaneously.

**Fig 2:**
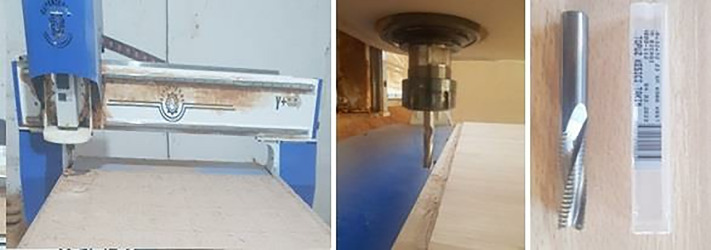
A three-axis CNC milling machine and CNC Tool.

A three-axis numerical control milling machine manufactured by Sepenta (Iran) with a 6-kW spindle and a maximum rotational speed of 32,000 rpm was used to perform the experimental tests (Fig 2). A carbide tool (Tideway, Turkey) with a diameter of 10 mm was employed (Fig 2).

Industrial beech wood was used for all experiments. Prior to machining, all samples were kiln-dried for one month until reaching a relative humidity of approximately 10%.

**Fig 3:**
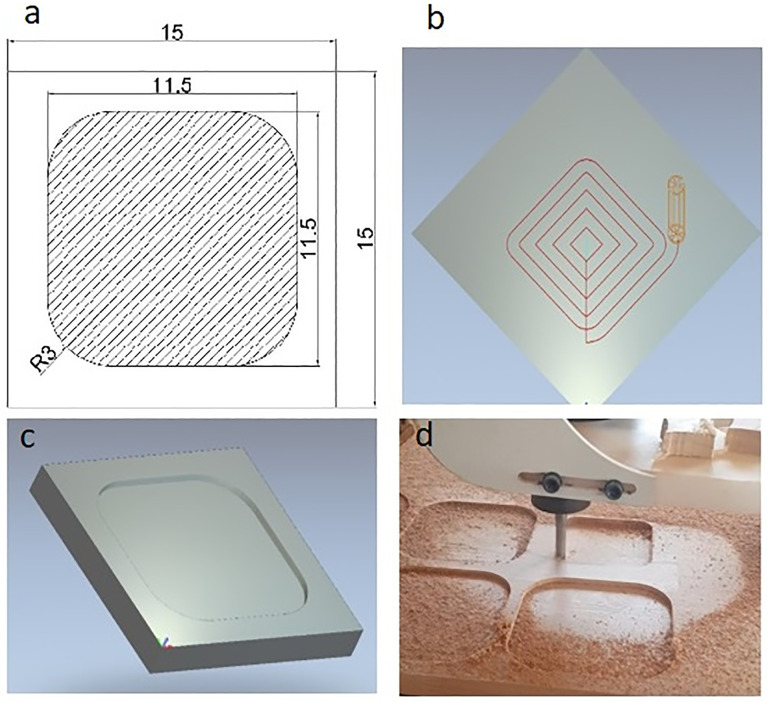
Machining sample. a) machinig area, b) tool path strategy, c) machined area (ArtCam), d) machined area (experimental test).

The sample dimensions were 15 × 15 cm, with a machining area of 11.5 × 11.51 cm (Fig 3a). An ‘Offset’ tool path strategy was adopted, in which the tool moves from the center of the machining area toward the periphery in step-over increments (Fig 3b). A 3D model of the sample and machining area was generated using ArtCam Pro 2008 software (Fig 3c), which converted the toolpath strategy into G-code and M-code for CNC execution. Fig 3d shows an example of the experimental CNC milling process. All machining was performed in the direction of the wood grain to maintain consistent cutting conditions across tests.

The assessment of noise exposure for CNC milling workers was performed in accordance with ISO 9612. The equivalent continuous sound pressure level (LAeq) was measured using a Casella CEL 450 sound level meter, set to A-weighting and slow response mode. The microphone was positioned at a height of 1.5 meters from the floor and at a horizontal distance of approximately 1 meter from the CNC machine, corresponding to the typical location of the operator’s ear during machine operation. Measurements were taken under normal operating conditions [22].

To optimize the developed NPL prediction model, a genetic algorithm (GA) was employed. GA is a stochastic search and optimization technique inspired by the principles of natural selection and genetics, widely used for solving complex, nonlinear, and multi-parameter optimization problems. In manufacturing research, GA has been successfully applied to machining parameter tuning in CNC turning centers [23], micro milling processes [24], and multi-pass milling optimization using hybrid GA-simulated annealing approaches [25].

In the GA framework, each machining parameter was encoded as a binary gene, with four genes forming a chromosome representing a potential solution. The machining parameters and the experimental setup data are summarized in Table 1.

**Table 1:** CNC milling condition.

CNC machine	A three-axis CNC machine, Sepenta (Iran)
Depth of cut $({𝐚}_{𝐩})$	3, 4, 5 (mm)
Feed rate $\left({𝐯}_{𝐖}\right)$	50, 100, 150 (mm/s)
Cutting speed $\left({𝐯}_{𝐜}\right)$	12000, 15000, 18000 (rpm)
Step over $\left({𝐬}_{𝐨}\right)$	3, 5, 7 (mm)
CNC tool	a carbide tool, Tideway (Turkey)
CNC tool diameter	10 (mm)
Tool Path strategy	Offset milling
Workpiece material	beech
Dimensions of workpieces	15*15 (cm)
Dimensions of machining area	11.5*11.5 (cm)
Sound level meter	Casella CEL 450
Sound level parameter	Equivalent sound pressure (LAeq)

## 3. Results and discussion

### 3.1. Design of experiments (DOE)

In order to investigation of the effect of the machining parameters including depth of cut, feed rate, cutting speed and step over on NPL the experimental test designed by Box-Behnken Design (=BBD).

BBD is a class of RSM which is the most popular second order design provide modelling of the NPL introduced by Arun et al. [26].

Table 2 shows the process variables used in the experiments.

**Table 2:** Process variables used.

Process variable	Notation	Unit	Limits		
Depth of cut	$a_{p}$	mm	3	4	5
Feed rate	$v_{W}$	mm/sec	50	100	150
Cutting speed	$v_{c}$	rpm	12000	15000	18000
Step over	$s_{o}$	mm	3	5	7

A BBD for this condition consists of 27 experiments. BBD matrix with actual variables and experimental values is shown in Table 3.

**Table 3:** Box-Behnken Design matrix, and experiment values.

Run Order	$a_{p}$	$v_{W}$	$v_{c}$	$s_{o}$	NPL (dB)
1	3	100	15000	3	99.5
2	4	100	15000	5	100.6
3	5	100	18000	5	103.8
4	4	150	15000	3	101.7
5	4	100	12000	7	99.6
6	3	150	15000	5	99.7
7	4	100	15000	5	100.1
8	4	100	15000	5	99.3
9	3	100	12000	5	98.5
10	4	100	12000	3	97.4
11	5	50	15000	5	99.3
12	4	50	15000	3	99.7
13	3	50	15000	5	97.4
14	4	50	12000	5	97.9
15	5	100	12000	5	100.7
16	4	150	12000	5	100.2
17	4	150	15000	7	101
18	5	150	15000	5	100.1
19	4	100	18000	7	98
20	5	100	15000	3	100.7
21	3	100	15000	7	99.3
22	4	100	18000	3	102.1
23	4	150	18000	5	103
24	4	50	18000	5	102.7
25	4	50	15000	7	99.1
26	3	100	18000	5	101.8
27	5	100	15000	7	100.5

According to NIOSH, an 8-h time-weighted average exposure limit of 85 dB and a 3-dB exchange rate were recommended. Table 3 shows that the amount of NPL at all of the experimental tests are higher than the standard value (NIOSH: 85dB). So it is necessary for all CNC wood workers to be entered in a hearing loss prevention program (HLPP) [18].

### 3.2. Analysis of variance (ANOVA)

The analysis of variance of experimental data was done to statistically analyse the relative significance of the machining parameters (Table 4). These parameters include depth of cut, feed rate, cutting speed and step over on response variable NPL. According to Table 4, amount of P-Value for $a_{p}.\;v_{W}$ and $v_{c}$ is lower than 0.05 so these parameters have a significant effect on NPL. Also, terms of $v_{c}^{\text{2}}$, and $v_{c}*s_{o}$ have similar conditions and are significant on NPL but other interactions have negligible effects.

**Table 4:** ANOVA table for noise pollution noise based on BBD.

Source	DF	Adj SS	Adj MS	F-Value	P-Value
Model	14	54.4590	3.8899	12.99	0.000
Linear	4	41.5902	10.3976	34.71	0.000
$a_{p}$	1	6.6008	6.6008	22.04	0.001
$v_{W}$	1	7.6800	7.6800	25.64	0.000
$v_{c}$	1	25.5454	25.5454	85.28	0.000
$s_{o}$	1	0.0844	0.0844	0.28	0.607
Square	4	10.7105	2.6776	8.94	0.002
$a_{p}^{2}$	1	1.0547	1.0547	3.52	0.090
$v_{w}^{2}$	1	0.7922	0.7922	2.64	0.135
$v_{c}^{2}$	1	8.4467	8.4467	28.20	0.000
$s_{o}^{2}$	1	1.4423	1.4423	4.82	0.053
2-Way Interaction	6	3.2327	0.5388	1.80	0.197
$a_{p}*v_{w}$	1	0.5625	0.5625	1.88	0.201
$a_{p}*v_{c}$	1	0.0100	0.0100	0.03	0.859
$a_{p}*s_{o}$	1	0.0000	0.0000	0.00	1.000
$v_{w}*v_{c}$	1	1.0000	1.0000	3.34	0.098
$v_{w}*s_{o}$	1	0.0025	0.0025	0.01	0.929
$v_{c}*s_{o}$	1	1.6577	1.6577	5.53	0.040
Error	10	2.9954	0.2995		
Lack-of-Fit	8	2.1354	0.2669	0.62	0.742
Pure Error	2	0.8600	0.4300		
Total	24	57.4544			

The results of ANOVA analysis show that cutting speed (V_c_), cutting speed squared (V_c²_), feed rate (V_w_) and depth of cut (a_p_) have a significant effect on the noise pollution level. These findings are consistent with the study of Wellenreiter et al., 2023, which showed that increasing cutting speed increases noise levels due to increased tool-material contact [21]. Also, Spinelli et al. (2016) investigated the effect of the number of tool contacts and lower speed on noise reduction, which is consistent with our results on the effect of depth of cut [6].

**Fig 4:**
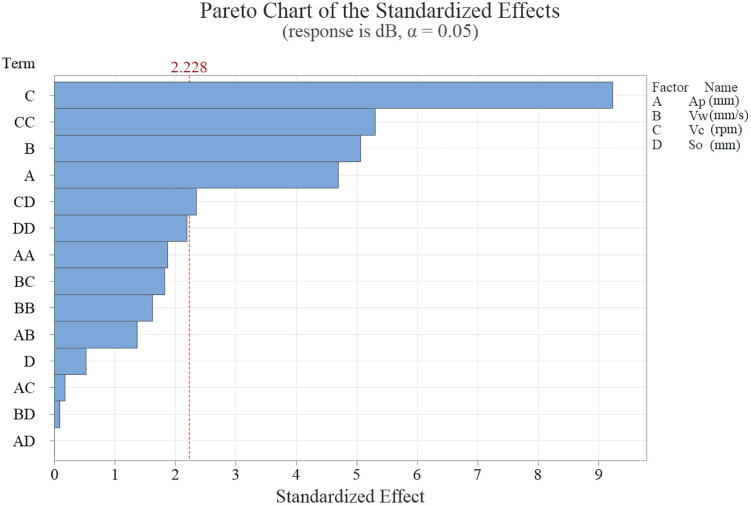
Pareto Chart of the standardized effects.

In Fig 4, the Pareto chart related to the effect of the main, square and interactions of parameters on the output parameter (NPL) is shown. Based on this diagram, with a confidence factor of 0.95, the main parameters have the greatest effect; C: cutting speed, CC: square of cutting speed, B: feed rate, A: depth of cut and CD: interaction of cutting speed and step over. In this graph, any bar that crosses the vertical line of 2.228 indicates that it affects the response variable (NPL). The mentioned number is calculated based on the confidence factor of 95%. In fact, the Pareto chart seeks to identify the factors whose total impact on the response variable constitutes more than 80% of the impacts. In other words, it seeks to determine 20 percent of the factors whose contribution to the changes of the response variable is equal to 80%.

As the results show, increasing the cutting speed has a strong effect on increasing the level of noise pollution. These findings are consistent with the study by Gholamian et al., who reported that the sound emission rate increased significantly with increasing cutting speed. This relationship was attributed to factors such as wood density, moisture content, and saw feed rate [27]. Furthermore, our findings on the effect of depth of cut are consistent with the results of Spinelli et al. 2016, who showed that reducing the number of tool collisions and lower speed can reduce noise levels [6].

**Fig 5:**
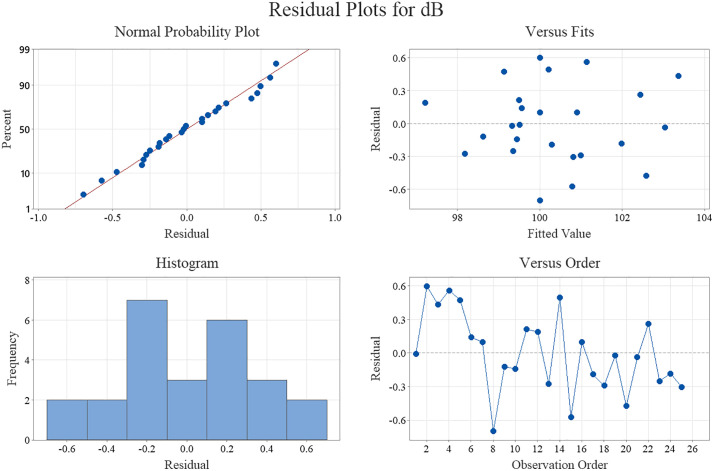
Residual plots for the regression model predicting NPL.

To assess the adequacy and assumptions of the developed regression model, a comprehensive residual analysis was conducted. The residual diagnostic plots presented in Fig 5 provide insight into the validity of the model assumptions. The normal probability plot indicates that the residuals closely follow a straight line, suggesting that they are approximately normally distributed. The residuals versus fitted values plot displays no discernible pattern, confirming the assumption of homoscedasticity and suggesting that the variance of the residuals remains constant across all levels of the fitted values. Additionally, the histogram of the residuals further supports the normality assumption, showing a symmetric distribution centered around zero. The residuals versus observation order plot reveals no systematic trends or autocorrelation, indicating the independence of residuals across the experimental runs. Collectively, these diagnostic plots validate the robustness of the regression model and confirm the suitability of the applied statistical approach.

**Fig 6:**
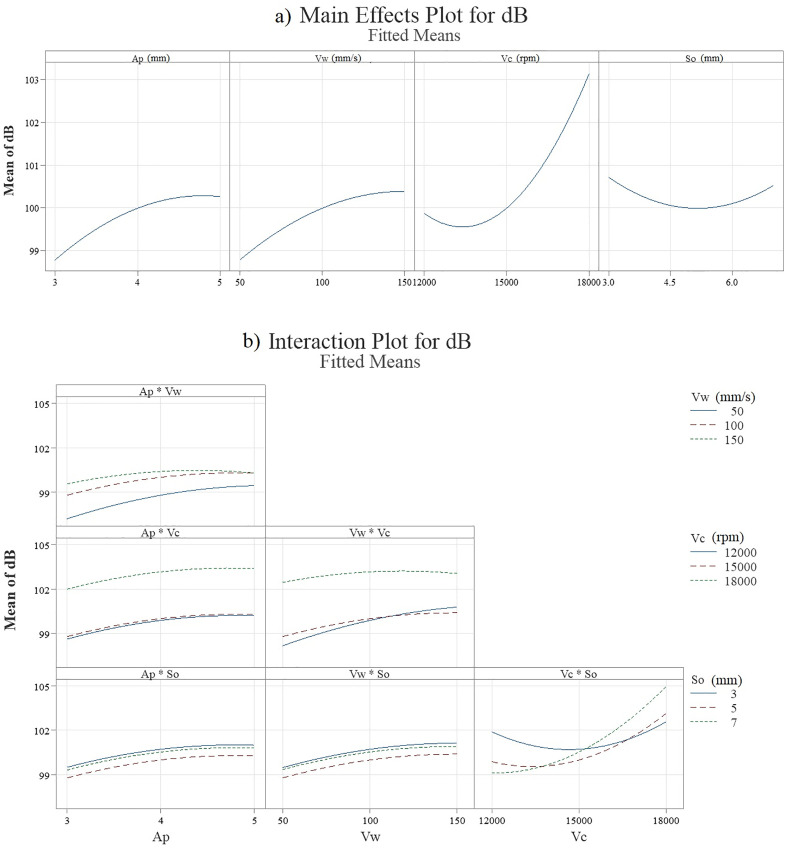
Indicates effect of main parameters (a) and their interaction (b) (fitted data).

Fig 6 indicates effect of main parameters and their interaction on NPL based on fitted mean derived from regression model. A main effects plot (Fig 6 a) is a plot of the mean response values at each level of a design parameter or process variable. This plot is used to compare the relative strength of the effects of various factors. In other words, the main effect is the effect of an independent variable ($a_{p}.\;v_{W}$,$\;v_{c}\;$or$\;s_{o}$) on a dependent variable (NPL) averaging across the levels of any other independent variables. According to Fig 5, change in$\;v_{c}$,$\;a_{p}\;$and$\;v_{W}\;$leads to sever changes of NPL. But$\;s_{o}\;$has less effect on the NPL (Fig 6 a). Also, in general, the amount of NPL increases with the increase of$\;v_{c}$,$\;a_{p}\;$and$\;v_{W}$. Fig 6 b shows interaction plot for NPL (fitted mean). Interactions occur when variables act together to impact the output of the process. Interactions plots are constructed by plotting both variables together on the same graph. Parallel lines: No interaction occurs. Nonparallel lines: An interaction occurs. The more nonparallel the lines are, the greater the strength of the interaction. As Fig 6 b shows there is sever interaction between$\;v_{c}\;$and$\;s_{o}\;$($v_{c}*s_{o}$).

### 3.3. Effect of machining parameters

**Fig 7:**
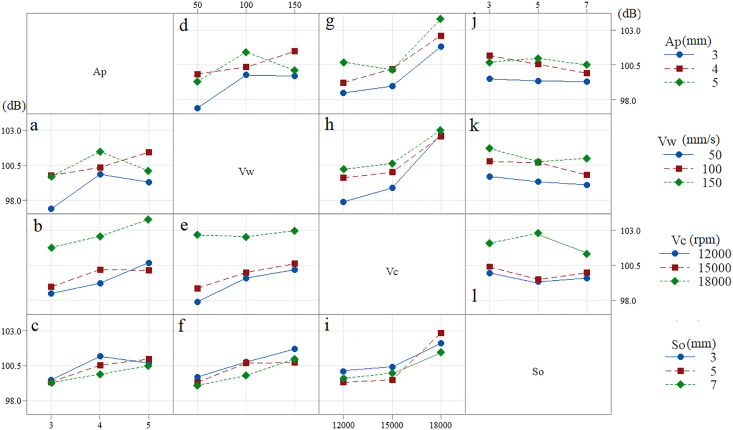
General effect of machining parameters on NPL.

Fig 7 shows the effect of machining parameters on NPL in one plot based on data means (raw data). By changing machining parameters amount of noise level changes from 97.4 to 103.8 dB (6.4 dB difference).

The noise level change from 97.4 dB to 103.8 dB indicates a significant influence of machining parameters in industrial environments. This result is comparable to the study of Krimpenis et al., 2016, which used genetic algorithm (GA) to optimize machining operations. They also found that fine-tuning of machining parameters can effectively reduce noise levels [28].

Fig 7 a, b and c indicate effect of depth of cut under different feed rate, cutting speed and step over condition. As the Fig 7 shows, with the increase of the depth of cut, under different machining conditions, the amount of NPL increases.

Therefore, in order to reduce the amount of NPL, the depth of cut should be reduced as much as possible. Effect of feed rate on NPL is shown in Fig 7 d, e and f. Similar results are observed with the results of depth of cut. Generally, as the feed rate increases, under different machining conditions, the amount of NPL increases. Fig 7 g, h and i illustrates effect of cutting speed on NPL. The results show that with the increase of cutting speed, the amount of NPL increases more intensively. Therefore, the cutting speed has a great effect on the amount of NPL especially at the cutting speed of 18000 rpm which the value of NPL increases strongly. The effect of the step over on the NPL value is different from other machining parameters. As Fig 7 J, k and l shows, changes in the step over do not have a great effect on the NPL value. Also, the general effect of step over on NPL is not clear. It can be seen that in some tests, increasing the step over leads to an increase in NPL, and in some tests, it leads to a decrease, and even in some tests, it does not change the NPL.

In the study of Gholamiyan et al., it was also found that increasing cutting speed resulted in a corresponding increase in noise levels. This relationship was attributed to the dynamics of the cutting process, where higher speeds create more vibrations and noise emission [27].

**Fig 8:**
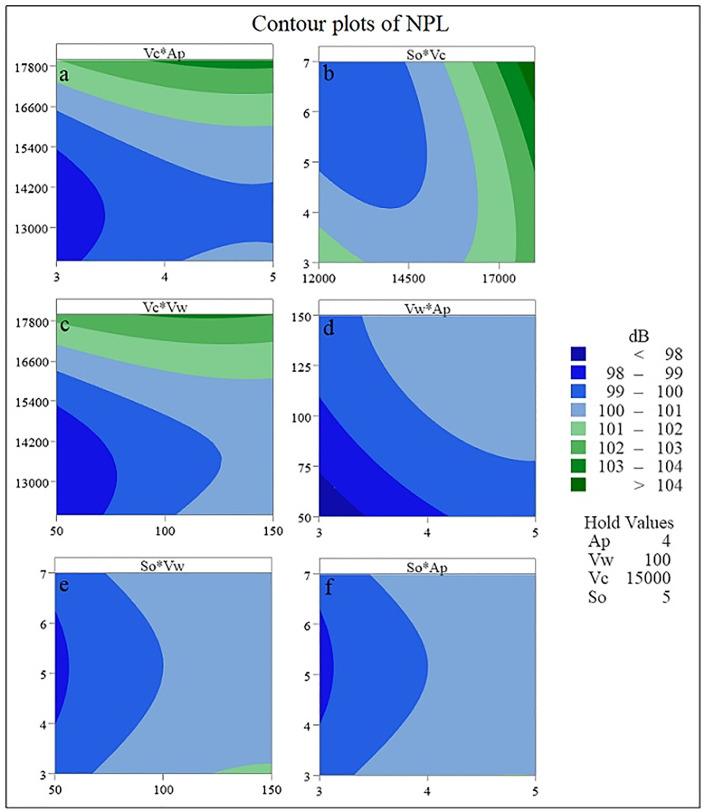
Contour plots of NPL.

Fig 8 shows contour plots obtained from BBD. Fig 8 shows the effect of different machining parameters on the amount of NPL as well as its maximum and minimum values areas. In the areas with dark blue color, the lowest amount of NPL has been created. In these areas, the noise level is less and it is more suitable for the health of the operator. By choosing the depth of cut value between 3 and 3.5 mm and the feed rate between 50 and 75 mm/s and the cutting speed of 15000 rpm and the step over of 5 mm, the lowest value (less than 98 dB) for NPL is obtained (Fig 8 d). As the color changes from dark blue to light blue, light green and finally dark green, the amount of NPL increases. In fact, the areas with dark green color have the worst conditions for the operator’s health in terms of exposure to loud noise. By choosing a cutting speed between 17000 and 18000 rpm, a step over between 6 and 7 mm and a feed rate value of 100 mm/s and a cutting depth of 4 mm, the highest value of NPL (more than 104 dB) is created (Fig 8 b).

### 3.4. Modelling

In this research BBD which is a class of RSM have been performed to predict noise pollution level in milling of beech wood. RSM is a combination of mathematical and statistical techniques which are useful in building the models and analysing the problems. The mathematical model of the response with regard to independent parameters can be predicted by employing the multiple regression analysis. In this method, number of experiments is reduced through design of experiment methods (DOE) [29].

According to the RSM, the quantitative form of relationship between the desired response and independent input variables is represented as Eq. (1):


$$Y=f(a_{p}.\;v_{W}.\;v_{c}.\;s_{o})$$ (1)

where Y is the desired response (NPL), f is the response function and$\;a_{p}$,$\;v_{W}$,$\;v_{c}\;$and$\;s_{o}\;$are the machining parameters. In order to prediction of NPL a second order polynomial response surface has been fitted into Eq. (2):


$$Y=b_{\text{0}}+\;\sum_{i=\text{1}}^{n}b_{i}x_{i}\sum_{i=\text{1}}^{n}b_{ii}x_{i}^{\text{2}}+\;\sum_{i=\text{1}}^{n-\text{1}}\sum_{j=i+\text{1}}^{n}b_{ij}x_{i}x_{j}$$ (2)

where Y is the corresponding response (NPL) and xi is the value of i th machining process parameter. Table 4 shows the data related to the model analysis and the effect of different parameters on the objective function. F-value of the model (12.99) indicates that the model is “significant” for NPL. Also, P-Value for the model is less than 0.05 (0.000) which indicates that the model terms are “significant”. Finally, the verification of the models has also been tested by the coefficient of R^2^. The R^2^ value (0.945) is high, close to 1, which is desirable.

The regression equation that has been obtained for response factors by using multiple regressions is:


$$\begin{array}{c} \text{NPL}\;=\;\text{127}.\text{1}\;+\;\text{5}.\text{49}\;a_{p}\;+\;\text{0}.\text{1297}\;v_{W}\;-\;\text{0}.\text{00517}\;v_{c}\;-\;\text{4}.\text{80}\;s_{o}\;-\;\text{0}.\text{469}\;a_{p}^{\text{2}}\;-\;\text{0}.\text{000162}\;v_{w}^{\text{2}}\;\\ \;\;\;+\;\text{0}.\text{000001}\;v_{c}^{\text{2}}\;+\;\text{0}.\text{1563}\;s_{o}^{\text{2}}\;-\;\text{0}.\text{00750}\;a_{p}*v_{w}\;-\;\text{0}.\text{000017}\;a_{p}*v_{c}\;+\;\text{0}.\text{001}\;a_{p}*s_{o}\;\\ \;\;\;-\;\text{0}.\text{000003}\;v_{w}*v_{c}\;-\;\text{0}.\text{00025}\;v_{w}*s_{o}+\;\text{0}.\text{000215}\;v_{c}*s_{o} \end{array}$$ (3)

### 3.5. Optimization

In order to optimize the developed model, genetic algorithm (GA) is utilized. GA is a class of search techniques inspired from the biological process of evolution by means of natural selection. They can be used to construct numerical optimization techniques that perform robustly on problem characterized by ill-behaved search spaces. The optimization problem of NPL is stated as minimizing the NPL which is subjected to a set of constraints. In the present investigation, the constrained optimization problem using GA is: “finding the optimal values of $\;a_{p}$, $\;v_{W}$, $\;v_{c}\;$ and $\;s_{o}$”. The optimization problem is minimizing of the NPL using the model given in Eq. (3) regarding to the following constraints:


$$\text{3}\leq s_{o}\leq \text{7},$$

$${\text{5}\text{0}\leq v}_{W}\leq \text{1}\text{5}\text{0},$$

$$\text{1}\text{2}\text{0}\text{0}\text{0}\leq v_{c}\leq \text{1}\text{8}\text{0}\text{0}\text{0},$$

$$\text{3}\leq s_{o}\leq \text{7}$$

In the GA, the genes are searching parameters, which are represented with finite length of binary codes, 0 and 1. The chromosomes are the strings of defining genes. Thus, the chromosome for the GA optimization in the present investigation consists of 4 genes corresponding to four searching parameters$\;a_{p}$,$\;v_{W}$,$\;v_{c}\;$and$\;s_{o}$. Each gene is represented by 20 bits of binary codes and hence a chromosome is of length 60 bits. The steps involved in the GA optimization in the present investigation are as follows:

### Step 1: generate an initial chromosome population randomly.

Step 2: decode the genes$\;a_{p}$,$\;v_{W}$,$\;v_{c}\;$and$\;s_{o}\;$of all chromosomes.

Step 3: evaluate the predicted values of NPL using the developed model (Eq. 3).

Step 4: determine the fitness of all chromosomes and obtain the maximum fitness (fitmax).

Step 5: if fitmax required fitness, then carryout following genetic operations:

a. Selection based on expected number control method,
b. Crossover, and
c. Mutation

**Fig 9:**
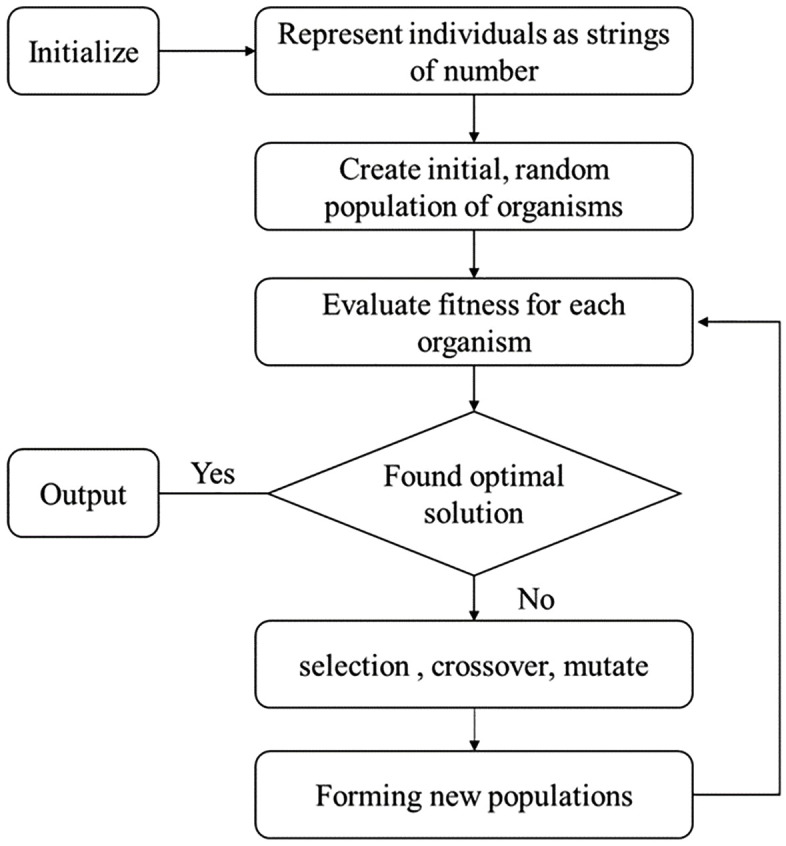
The flowchart of the genetic algorithm.

To generate new chromosome population and go to step 2. Else stop. Fig 9 shows the flowchart of the genetic algorithm.

MATLAB (Math Works Corporation, 2005) has been used to develop the GA code. The following GA parameters have been selected to obtain the best possible solutions:

maximum number of generations  = 100;
total string length  =  60;
no. of chromosomes  =  50;
cross over = Two points;
crossover probability  =  0.8;
mutation  =  Two bits;
mutation probability  =  0.003

The levels of input process parameter have been fed to the GA and the values of cutting conditions have been predicted for minimum NPL. The minimum values of NPL have been predicted by the GA with respect to the ranges of machining parameters. The corresponding optimal machining conditions are presented in Table 5. From the optimization results of GA, it is seen that the minimum NPL value is 96.2 dB that has a good agreement with result of experiment test (95.8 dB). Furthermore, it is observed that the optimal conditions for achieving better NPL can be determined using GA. It is really beneficial to improve performance of eco-friendly CNC wood machining.

**Table 5:** Output values of GA for chosen machining parameters.

$a_{p}\;$(mm)	$v_{W}\;$(mm/s)	$v_{c}\;$(rpm)	$s_{o}\;$(mm)	Predicted NPL	Actual NPL
3.2	58	12730	6.4	96.2	95.8

## 4. Conclusion

Exposure to the high level of noise pollution in CNC wood machining can case variety of adverse health disorders. In this research CNC milling of beech wood is carried out. Effect of machining parameters including depth of cut, feed rate, cutting speed and step over was investigated and the following results were observed:

- CNC wood machining leads to unsafe operator’s work environment. In 100% of the tests, the amount of noise pollution level (NPL) is more than the standard value (in comparison with NIOSH: 85 dB). Therefore, it is necessary for all CNC wood workers to be entered in a hearing loss prevention program (HLPP).
- According to ANOVA and Pareto Chart machining parameters including; cutting speed, square of cutting speed, feed rate, depth of cut and interaction of cutting speed and step over have significant effect on NPL respectively.
- Machining parameters have a very important effect on the amount of NPL. The highest value is 103.8 dB and the lowest value is 97.4 dB.
- In general, increasing depth of cut, feed rate and cutting speed leads to increasing NPL. Cutting speed has more effect than others. The step over does not have a significant effect on the NPL.
- The regression equation based on the response procedure method predicts well the amount of NPL based on the machining parameters. This equation was validated based on statistical principles.
- It is observed that the optimal conditions for achieving better NPL can be determined using genetic algorithm. It is really beneficial to improve performance of eco-friendly CNC wood machining.
- By choosing $\;a_{p}=\;\text{3}\;.\text{2}\;mm\;.v_{W\;}=\text{58}\;\frac{mm}{s}.\;v_{c}=\text{12730}\;rpm\;$ and $\;s_{o}=\text{6}.\text{4}\;mm$, the amount of predicted NPL is reduced to 96.2 dB. This prediction tested and validated by experimental test (95.8 dB).

This study showed that optimizing machining parameters using genetic algorithms can lead to a significant reduction in noise levels in wood machining. These findings are consistent with previous studies and indicate that the use of advanced optimization methods can bring improvements in the industrial workplace [ 6,28,30].

The findings from this study underscore the critical need for optimizing machining parameters in CNC wood milling to mitigate noise pollution levels (NPL). Given that all experimental tests exceeded the NIOSH standard of 85 dB, it is essential for woodworking facilities to implement hearing loss prevention programs (HLPP) for workers. By adopting the optimal parameters identified in this research—such as a depth of cut of 3.2 mm, feed rate of 58 mm/s, and cutting speed of 12,730 rpm—companies can significantly reduce NPL, thereby enhancing workplace safety and employee well-being. Moreover, the application of Response Surface Methodology (RSM) provides a robust framework for continuous improvement in CNC operations. This study’s approach can serve as a model for other machining processes, helping industry practitioners understand the interactions between various parameters and their collective impact on operational outcomes.

Future studies could build upon the current findings by investigating the effects of floor vibrations and surface settlement in larger-scale workshops or environments with variable dynamic loads. Such factors may significantly influence the accuracy of noise level assessments and system response. Additionally, integrating targeted noise reduction strategies including sound insulation, vibration damping, and the optimization of machining parameters may offer a more comprehensive and effective approach to minimizing noise pollution levels (NPL) in CNC wood milling operations.
